# Design and Optimization of In Situ Gelling Mucoadhesive Eye Drops Containing Dexamethasone

**DOI:** 10.3390/gels8090561

**Published:** 2022-09-02

**Authors:** Boglárka Szalai, Orsolya Jójárt-Laczkovich, Anita Kovács, Szilvia Berkó, György Tibor Balogh, Gábor Katona, Mária Budai-Szűcs

**Affiliations:** 1 Institute of Pharmaceutical Technology and Regulatory Affairs, Faculty of Pharmacy, University of Szeged, Eötvös Str. 6, H-6720 Szeged, Hungary; 2 Department of Pharmacodynamics and Biopharmacy, Faculty of Pharmacy, University of Szeged, Eötvös Str. 6, H-6720 Szeged, Hungary; 3 Department of Chemical and Environmental Process Engineering, Budapest University of Technology and Economics, Műegyetem Quay 3, H-1111 Budapest, Hungary

**Keywords:** corneal-PAMPA, cyclodextrin, factorial design, ophthalmic delivery, penetration, poloxamer 407

## Abstract

Poor bioavailability of eye drops is a well-known issue, which can be improved by increasing the residence time on the eye surface and the penetration of the active pharmaceutical ingredient (API). This study aims to formulate in situ gelling mucoadhesive ophthalmic preparations. To increase the residence time, the formulations were based on a thermosensitive polymer (Poloxamer 407 (P407)) and were combined with two types of mucoadhesive polymers. Dexamethasone (DXM) was solubilized by complexation with cyclodextrins (CD). The effect of the composition on the gel structure, mucoadhesion, dissolution, and permeability was investigated with 3^3^ full factorial design. These parameters of the gels were measured by rheological studies, tensile test, dialysis membrane diffusion, and in vitro permeability assay. The dissolution and permeability of the gels were also compared with DXM suspension and CD-DXM solution. The gelation is strongly determined by P407; however, the mucoadhesive polymers also influenced it. Mucoadhesion increased with the polymer concentration. The first phase of drug release was similar to that of the CD-DXM solution, then it became prolonged. The permeability of DXM was significantly improved. The factorial design helped to identify the most important factors, thereby facilitating the formulation of a suitable carrier for the CD-DXM complex.

## 1. Introduction

The eye is one of our most important sensory organs. Its disease or injury can impair the quality of life. Bypassing the complex defense mechanisms of the eye and thus achieving effective ocular drug delivery poses challenges for pharmaceutical technologists.

Eye drops are commonly used to locally treat ocular diseases. The disadvantage of topical drug administration is the poor bioavailability. Permeability through the cornea is limited as eye drops are eliminated from the site of application within minutes due to overflow, nasolacrimal drainage, blinking, and increased tear production after instillation [1,2]. Eye drops should be frequently administered because of rapid elimination, which reduces patient compliance [3].

The tear film and the cornea are composed of hydrophilic and lipophilic layers; therefore, the absorption of moderately lipophilic compounds is favored. These compounds are often poorly water-soluble, so they should be formulated as suspensions. Although the bioavailability of topical formulations is generally less than 5% [4], it is more advantageous than those administered systemically. This is because a lower dose might be sufficient to achieve a therapeutic effect causing little to no systemic effects, and the effect develops rapidly [1].

Topical preparations should reach the site of action by transcorneal penetration into the interior segment of the eye. As bioavailability is determined by the absorption and elimination rate of the API [5], bioavailability can be increased by reducing elimination and increasing absorption. This can be achieved by, for instance, better corneal penetration and longer corneal residence.

In situ gels and applying mucoadhesive polymers are effective in increasing the residence time. They act as hydrogels that convert into a gel under physiological conditions. The most common stimuli are ionic interactions (e.g., gellan gum [6]), temperature (e.g., poloxamers [7], cellulose derivates [8]), and pH (e.g., carbomers [9]). In situ gels are liquid at non-physiological conditions and room temperature, so they are easy to apply and they form a gel after contact with the eye, allowing the composition to remain on the eye surface longer [1,3].

P407 is a type of polymer that can form thermosensitive in situ gels; it is biocompatible, easy to sterilize, and suitable for carrying small and large molecules as well. The temperature of the sol–gel transition and drug release profile can be precisely controlled by modifying the composition. Due to all these characteristics, its use in topical ophthalmic preparations is advantageous [3].

DXM is used to treat ophthalmic inflammations that occur in response to allergies or trauma to the eyes (e.g., surgery) [10,11]. Its water-solubility is low, and the amount of API required to achieve the therapeutic effect is not soluble in water. DXM-containing eye drops are mainly marketed as suspensions.

In general, there are several problems with ocular suspensions including inhomogeneity, sedimentation, aggregation of suspended particles, and re-suspensibility. In addition, particles larger than 1 μm might irritate the eye [12]. Ensuring their physical stability and the limits of sterilization methods pose challenges in their formulation. For proper dosing, suspensions should be well shaken prior to administration, but patients might have trouble with it. The amount of drug required to achieve the therapeutic effect is relatively high due to poor bioavailability, which makes suspension eye drops expensive [12,13].

Reformulating the eye drops as solutions may be the solution for these problems. The use of API derivates with better water solubility is a commonly used method. Drugs with both lipophilic and hydrophilic properties penetrate through the cornea the best; however, increasing the water solubility reduces the lipophilicity of the molecule, thus reducing transcorneal penetration [13].

CDs and their derivates are ring-shaped oligosaccharides with a hydrophilic outer surface and hydrophobic inner cavity. Inclusion complexes form when a lipophilic drug or a part of it fits in the cavity of the CD and forms secondary bonds with it. The structure of CDs allows the solubilization of poorly water-soluble drugs while preserving their lipophilicity, thus their permeability [13,14]. CD complexation can increase the penetration and absorption of drugs through the cornea by delivering hydrophobic drug molecules directly to the cornea. The inclusion complex cannot penetrate through the lipophilic barriers, the drug must be released from the complex to enter the eye through the cornea [15]. The use of CDs, however, does not always mean that penetration will increase. When CD is added to a suspension, the penetration of the drug across biological membranes initially increases as the amount of CD rises. After dissolving all drug molecules, increasing the amount of CD leads to a decrease in permeability. If more than the least necessary amount of CD is used, re-complexation of the drug is likely to occur, thus reducing the free fraction capable of penetration [12,15].

This work aimed to formulate in situ gelling eye drops that allow application as a liquid eye drop, then allow gelation to occur after distribution on the eye surface. An additional aim was to increase the mucoadhesion of the poloxamer-containing eye drops and to increase the water solubility of the API. Complex polymer systems with P407, zinc hyaluronate (ZnHA), and hydroxypropyl methylcellulose (HPMC) matrix were formulated. The formation of the gel matrix (gelling temperature, gelling time, gel strength) was investigated by means of rheological studies; mucoadhesivity was analyzed by the tensile test (adhesive work and force), and finally, the drug release behavior and permeability of DXM were investigated by in vitro models (dialysis bag method and corneal-PAMPA model). In order to analyze the possible effects of the components and their interactions, factorial design was applied (3^3^ full factorial design), which could help to choose the optimal composition(s).

## 2. Materials and Methods

### 2.1. Materials

(2-Hydroxypropyl)-beta-cyclodextrin (DS∼4.5; HPBCD), (2-Hydroxypropyl)-gamma-cyclodextrin (DS∼4.5; HPGCD), and Heptakis (2,6-di-O-methyl)-beta-cyclodextrin (content > 35%; DS∼14; DIMEB) were obtained from Cyclolab R&D Ltd. (Budapest, Hungary). Sulfobutylether-beta-cyclodextrin (DS∼2; SBEBCD) was provided by Sanofi-Aventis Ltd. (Paris, France). Kolliphor^®^ P 407 (oxyethylene 71.5–74.9%; P407) was purchased from Sigma-Aldrich (St. Louis, MO, USA). Hydroxypropyl methylcellulose (Methocel F4M; HPMC) was kindly supplied by Colorcon (Dartford, UK). DXM was obtained from Pharmacia and Upjohn Company LLC. Zinc-hyaluronate was kindly donated by Gedeon Richter Plc. (Budapest, Hungary).

Simulated tear fluid (STF) of pH = 7.4 was prepared by dissolving 6.78 g/L NaCl, 2.18 g/L NaHCO_3_, 0.084 g/L CaCl_2_ × 2H_2_O, 1.38 g/L KCl in distilled water, pH was adjusted with 0.1 M HCl [16]. Components of the STF were analytical grade and purchased from Sigma Aldrich Co. Ltd. (Budapest, Hungary). Mucin from porcine stomach (Sigma Aldrich Co. Ltd., Budapest, Hungary) was applied to prepare mucin dispersion.

### 2.2. Preformulation Studies (Phase Solubility Test)

Solubility studies were based on the Higuchi and Lach method [17]. An excess amount of DXM was added to 1.5 mL of purified water containing different concentrations of HPBCD, HPGCD, SBEBCD, and DIMEB (0–150 mM). The suspensions were stirred with a magnetic stirrer for 24 h at 25 °C. After sedimentation, supernatants of the suspensions were filtered through a 0.22 μm pore size membrane filter (Millex-HV Syringe Driven Filter Unit, 0.22 μm, PVDF Durapore, Millipore, Bedford, MA, USA). The DXM concentration was measured with UV-Vis spectrophotometry (Unicam UV/Vis Spectrometer, ATI Unicam, Cambridge, UK) at 242 nm.

The phase-solubility diagrams were achieved by plotting the solubility of DXM versus the concentration of the CD derivates. *K*_1:1_ was calculated from phase-solubility profiles via the following equation (Equation (1)) [18]:

(1) $$K_{1:1}=\frac{Slope}{S_{0}\left(1-Slope\right)},$$

where *S*_0_ is the intrinsic solubility of the drug, in this case, the water-solubility when CD concentration is 0 mM [19].

According to Loftsson et al. [18], the solubilizing efficiency of CDs is more important than the absolute value of *K*_1:1_. The solubilizing efficiency, also referred to as the complexation efficiency (CE), provides information on the ratio of the free CD concentration to that in the complex (Equation (2)) [18,19]:

(2) $$\mathrm{CE}=\frac{Slope}{1-Slope}$$

The presence of commonly used pharmaceutical excipients, for instance, polymers, buffer salts, and preservatives affect the value of *S*_0_, therefore, CE is more reliable than *K*_1:1_, as it is independent of *S*_0_ [18,19].

### 2.3. Methods

#### 2.3.1. Design of Experiments

Factorial designs are part of the quality-by-design approach that help understanding not just the effect of variables, but also the interactions between them [20]. The effects of the concentration of P407, ZnHA, and HPMC were determined according to a 3^3^ full factorial design model. The labeling used in the discussion of the response surface equations and the values for −1, 0, and +1 levels of the independent variables are displayed in Table 1. The dependent variables of the factorial design were: gelling temperature (°C), gelling time (min), storage modulus (G′; Pa), peak force (mN), and work of adhesion (mN.mm) related to the mucoadhesion study, release efficiency in 6 h ((Q)-6 h;%), in vitro release rate (IVRR; μg/mL), permeability (Pe; ×10^−6^ cm/s), and flux (J; ×10^−6^ mol/cm^2^·s) measured by dialysis membrane diffusion and corneal-PAMPA (Parallel Artificial Membrane Permeability Assay) methods.

The results of the experiments were evaluated with Statistica for Windows 13.5. (StatSoft Inc., Tulsa, OK, USA) software. The confidence interval was 95% and the alpha value indicating significant factors was 0.05. The unnecessary determinants were eliminated from the equations to increase the fitting accuracy by maximizing the adjusted R^2^ values. The whole data generated by the software and the adjustments are presented in the Supplementary Materials (Table S1).

#### 2.3.2. Sample Preparation

The first step of sample preparation was dissolving ZnHA in distilled water, then refrigerating it overnight. HPMC was dissolved, in case of samples containing it, and also refrigerated. Thermosensitive ocular gels were prepared by the modified cold method [21]. P407 was gradually added to the polymer solution while stirring at 4 °C. The samples were refrigerated overnight to achieve a clear solution. HPBCD was dissolved in the formulations, then 10 mg of DXM was added and sonicated until complete dissolution. The amount of the polymers used in the samples is presented in Table 1.

#### 2.3.3. Optical Test

The optical parameters (transmittance, refractive index) were measured by UV-Vis spectrophotometry (Unicam UV/Vis Spectrometer, ATI Unicam, Cambridge, UK) in the wavelength range between 200–800 nm and by an Abbe-type refractometer (Refractometer RL3, PZO, Warsaw, Poland). Aqueous solution of the polymers alone, their combination, and S27 of the factorial design were studied. The concentration of the polymers was the +1 level value in each case.

#### 2.3.4. Rheological Study

Rheological measurements were performed with a Physica MCR 302 Modular Compact Rheometer (Anton Paar, Graz, Austria). A cone and plate type measuring device was applied (diameter: 25 mm, cone angle: 1°, gap height: 0.05 mm). To measure the gelling temperature of the samples, the temperature was increased from 15 to 40 °C with a 1 °C/min heating rate. The measurement was carried out at constant frequency (1 rad/min) and constant strain (1%). The point of gelation was considered as the crossover of the storage modulus (G′) and the loss modulus (G″) curves. Directly after the measurement of gelling time, frequency sweep tests were performed to characterize the viscoelasticity of the samples. The tests were carried out at 35 °C with a strain value of 1%, which was within the linear viscoelastic region of the gels. G′ was registered at the angular frequency range of 0.1–100 rad/s. Gelling time was determined at 35 °C at a constant frequency (1 rad/min) and constant strain (1%).

#### 2.3.5. Mucoadhesivity (Tensile Test)

The measurement of the mucoadhesion was carried out with a TA.XT plus Texture Analyzer (Stable Micro Systems Ltd., Surrey, UK). The instrument was equipped with a 5 kg load cell. A mucoadhesion test rig was used, which consists of a tissue holder, where a filter paper disc can be placed. The filter paper was wetted with 50 μL of 8% (*w/w*) mucin dispersion to imitate the in vitro mucosal surface. The mucin dispersion was prepared with STF (pH = 7.4). The test rig was placed in a beaker filled with water to provide 35 °C during the measurements. The cylinder probe with a 10 mm diameter fits in the hole on the upper part of the tissue holder, therefore, it can get in contact with the mucous surface. 20–20 mg of the samples were applied onto the lower end of the probe. The probe was lowered to the artificial mucosal surface, and a 2500 mN preload was used for 3 min. Then the probe moved upwards at 2.5 mm/min speed until the adhesive bond was broken. Five parallel measurements were carried out. The work of adhesion (mN mm) and the adhesive force (mN) were used to characterize the mucoadhesive behavior of the formulations [22,23].

#### 2.3.6. In Vitro Drug Release Study

The dialysis bag method was applied to study the in vitro drug release of the samples. Zellutrans/Roth cellulose dialysis membrane tubes (10 mm wide, 6.37 mm diameter, MWCO: 12,000–14,000 D) were filled with approximately 600 μL of the samples. The exact sample mass was used for the calculations. The tubes were closed with Spectra/Por^®^ Closures (Spectrum Laboratories, Inc., Rancho Dominguez, CA, USA). From each composition of in situ gels, 3 replicates were used and placed into 25 mL of STF tempered at 35 °C while stirring continuously with a magnetic stirrer. Sink condition was ensured during the experiments. During the diffusion study, nine sampling times were used (15, 30, 45, 60, 120, 180, 240, 300, and 360 min) by removing 1 mL from the acceptor phase and replacing it with fresh STF. DXM suspension and DXM-CD solution without any polymer were used as references. The quantitative measurement of DXM was performed by HPLC (Shimadzu Nexera X2 UHPLC, Kyoto, Japan), equipped with a C18 reverse-phase column (Phenomenex Kinetex C18, 2.6 μm, 100 Å, 150 × 4.6 mm, Phenomenex, Torrance, CA, USA). Gradient elution was used for the separation with the following program: time (min)/% of acetonitrile: 0/35, 4.0/60, 4.01/35, 6.0/35. A 5 μL volume of samples was injected and analyzed at a wavelength of 240 nm. The flow rate was 1 mL/min, and the column temperature was 40 °C. The time of analysis was 6 min and the retention time of DXM was 3.2 min.

#### 2.3.7. In Vitro Corneal-PAMPA

The previously reported corneal-PAMPA method was applied for the measurement of in vitro transcorneal permeability [23]. DXM suspension, DXM-CD solution, and the in situ gelling formulations of the factorial design were used as donor samples.

The lipid membrane was prepared as follows: phosphatidylcholine (16 mg) was dissolved in a solvent mixture (70% (*v/v*) hexane, 25% (*v/v*) dodecane, 5% (*v/v*) chloroform), then each well of the donor plate (MultiscreenTM-IP, MAIPN4510, pore size: 0.45 mm, Millipore, Bedford, MA, USA) was coated with 5 μL of the lipid solution. Afterward, the solvent mixture was evaporated to form a phosphatidylcholine lipid membrane containing 10.67% (*w/v*) phosphatidylcholine. The donor plate was fitted into the acceptor plate (Multiscreen Acceptor Plate, MSSACCEPTOR, Millipore, Bedford, MA, USA). Each well of the acceptor plate contained 300 μL PBS solution (pH 7.4), and 150 μL PBS solution was pipetted onto the lipid membranes. The donor plate was covered with wet tissue paper and a plate lid to avoid evaporation. The plates were incubated for 4 h at 35 °C. The DXM concentration in the acceptor and donor phase was measured by the HPLC method (described in Section 2.3.6). Test solutions from PAMPA experiments were prepared in 96-well plates and sealed before injection. Three replicates were measured for each sample. The effective permeability and membrane retention of DXM were calculated according to the following equation (Equation (3)) [24]:

(3) $$P_{e}=-\frac{2.303\cdot V_{A}}{A\left(t-\tau_{SS}\right)}\cdot \log\left[1-\frac{c_{A}\left(t\right)}{S}\right]\;,$$

where *P_e_* is the effective permeability coefficient (cm s^−1^), *A* is the filter area (0.24 cm^2^), *V_A_* is the volume of the acceptor phase (0.3 mL), *t* is the incubation time (s), *τ_SS_* is the time to reach steady-state (s), *C_A_(t*) is the concentration of the compound in the acceptor phase at time point *t* (mol cm^−3^), *S* is the free DXM content in the donor phase.

#### 2.3.8. Statistical Analysis

The statistical analysis of the factorial design was performed by Statistica for Windows (version 13.5, StatSoft Inc., Tulsa, OK, USA). In vitro drug release and corneal-PAMPA parameters were compared using one-way ANOVA followed by Dunnett’s test with GraphPad Prism 5.0 software (GraphPad Software, Inc., San Diego, CA, USA). A level of *p* ≤ 0.05 was taken as significant, *p* ≤ 0.01 as very significant, and *p* ≤ 0.001 as highly significant.

## 3. Results

### 3.1. Preformulation Studies

In the preformulation studies, the solubility of DXM in water (mg/mL) was determined and phase solubility plots were recorded with the different CD derivatives. In this study, four different CD derivatives were tested: HPBCD [25,26,27], HPGCD [26,27], DIMEB [27], and SBEBCD [27,28], which can be found in the literature on ophthalmic formulations. One CD molecule usually forms an inclusion complex with one drug molecule. The stability constants (*K*_1:1_) of complexes is a tool to compare the affinity of drugs to CDs and their derivates, whereas CE shows the concentration ratio between complexed and non-complexed CDs [18,19]. Linear A_L_ type diagrams with a slope value less than unity suggest that one drug molecule forms a complex with one CD [29]. Based on the phase solubility curves, the formation of 1:1 complexes can be observed in the investigated concentration range. The figure showing the phase solubility curves was inserted into the Supplementary Materials as Figure S1. The slope of the linear function, the *K*_1:1_, and CE values are displayed in Table 2.

To improve the bioavailability of poorly water-soluble drugs, a *K*_1:1_ value between 200 and 5000 M^−1^ is favored [30]. In our case, HPGCD presented the highest *K*_1:1_ value while SBEBCD had the lowest. As only free drug molecules can penetrate through the cornea, the drug concentration in the gel decreases, while the CD content is constant. Thereby, *K*_1:1_ of the complex affects the drug penetration. The higher the *K*_1:1_ value, the lower the drug penetration rate is. The *K*_1:1_ value was in the lower range for HPBCD and SBEBCD, but the CE of HPBCD was higher, so it was proved to be ideal for the solubilization of DXM.

The optimal amount of CD used in the formulations can be decided based on the results of the phase solubility study as well. The lowest CD concentration that solubilizes the entire drug dose ensures the highest permeation through biological membranes. Although more CD than necessary decreases permeability, it is advised to include small excess to prevent precipitation during storage. In the formulations of the factorial design, 94.4 mM HPBCD was incorporated to solubilize 0.1% DXM, which is the therapeutic concentration of DXM in marketed ophthalmic formulations.

### 3.2. Optical Test

Topically applied ocular medications should not cause any visual disturbances, as it may reduce patient compliance [31]. To evaluate the effect of the components in the eye drops, transmittance and refractive index were determined.

Spectral transmittance curves of the measured samples are presented in the Supplementary Materials (Figure S3). The curves suggest that none of the polymers reduced the transmittance of visible light; however, in the case of samples containing both P407 and ZnHA, the transmittance was slightly decreased (>90%). Notably, S27 blocked out the UV light below 280 nm, while still being transparent in visible light.

The cornea and the tear film together form one of the refractive media of the eye. Both consist of multiple layers with different refractive power. The average refractive index of the precorneal tear film is about 1.337–1.482 [32]. The refractive index values of the samples are presented in Table 3, and were within the physiological range, except for the ZnHA and HPMC solutions.

### 3.3. Analysis of Gel Structure

Thermosensitive in situ gelling eye drops should turn into gel at the temperature of the eye surface (34–35 °C [33]), but not at room temperature, thereby eye drops are easily applied as free-flowing liquids, and they can spread on the eye surface, then transform into gels due to body heat. As the gelation occurs, the viscosity increases, resulting in an elongated residence time.

Ideally, the eye drops should go through gelation between 28–34 °C. The gelation process should be completed within 5 min, as the tear layer renews every 5–6 min with an average tear turnover rate of approximately 15%/min [34]. Additionally, after the G′ value begins to increase, the gelation should be completed in an instant. Studies reported that different excipients and APIs might influence the gelling temperature [35]. The addition of polymers to the poloxamer solution might change the gelling characteristics and the final gel structure of the formulations, e.g., due to the formation of the polymer–polymer interaction or the viscosity changes of the initial liquid solutions.

The relationship between the composition (x_1_–x_3_ are listed in Table 1) and the properties of the gels (gelling temperature (y_1_), gelling time (y_2_), and G′ (y_3_)) are described with Equations (4)–(6). Significant factors are highlighted with bold-faced letters in the equations.

(4) $$y_{1}=28.44-5.25x_{1}-0.70x_{1}{}^{2}-0.52x_{2}-0.42x_{3}+0.53x_{1}x_{2}+0.62x_{1}x_{3}+0.62x_{1}{}^{2}x_{3}+0.28x_{1}{}^{2}x_{3}{}^{2}-0.45x_{2}{}^{2}x_{3}{}^{2}$$

(5) $$y_{2}=2.26-2.41x_{1}-0.83x_{1}{}^{2}-0.072x_{2}{}^{2}+0.23x_{1}x_{2}{}^{2}+0.23x_{1}{}^{2}x_{2}{}^{2}+0.17x_{1}x_{3}-0.12x_{1}x_{3}{}^{2}+0.20x_{1}{}^{2}x_{3}-0.12x_{1}{}^{2}x_{3}{}^{2}-0.17x_{2}{}^{2}x_{3}{}^{2}$$

(6) $$y_{3}=4651.65+6020.54x_{1}-2367.49x_{1}{}^{2}-276.33x_{2}-82.02x_{2}{}^{2}-277.94x_{1}x_{2}-89.30x_{1}x_{2}{}^{2}-100.15x_{1}x_{3}+71.79x_{1}x_{3}{}^{2}+117.66x_{1}{}^{2}x_{3}-155.24x_{2}x_{3}{}^{2}$$

**Figure 1:**
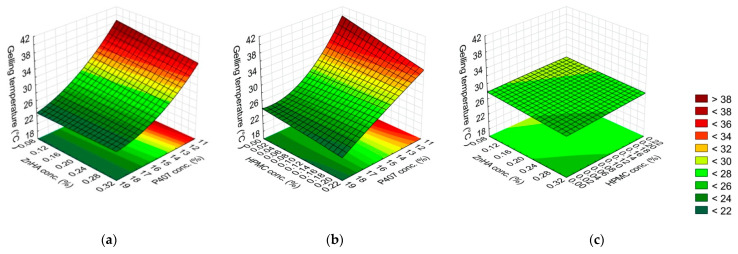
Effect of the composition on the gelling temperature: (**a**) P407 and ZnHA concentrations, (**b**) P407 and HPMC concentrations, (**c**) ZnHA and HPMC; (the 3rd factor is at 0 level in each case).

The gelling temperature was determined by only the P407 concentration (linear (L) and quadratic (Q) factor), the other polymers were not significant in this aspect. The negative coefficients of the significant factors indicate an inverse relationship, meaning the increase of P407 concentration results in a decrease in the temperature of the sol–gel transition. In our case, only P407 affects the gelation temperature but other polymers do not (Figure 1, Table 4).

**Figure 2:**
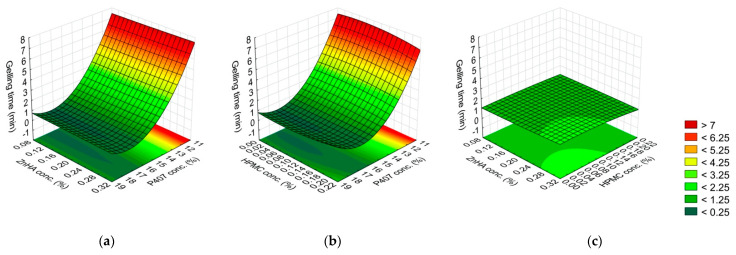
Effect of the composition on the gelling time: (**a**) P407 and ZnHA concentrations, (**b**) P407 and HPMC concentrations, (**c**) ZnHA and HPMC; (the 3rd factor is at 0 level in each case).

Regarding the gelling time, the effect of the P407 concentration was highly significant (*p* ≤ 0.001). The higher the P407 concentration, the shorter the process of gelling was. Other significant factors were the interaction of P407 (L, Q) and ZnHA (Q) concentrations, and P407 (Q) and HPMC (L) concentrations. These factors were directly proportional to the gelling time, while the interaction between ZnHA (Q) and HPMC (Q) concentrations was inversely proportional. This means that combining P407 with other polymers does not alter the gelation temperature but may slow down the gelation process at the given temperature. However, it should be considered that the interaction of the other two polymers can be influential, for our case, ZnHA and HPMC together had a positive effect, i.e., they accelerate the gelation (Figure 2, Table 4).

**Figure 3:**
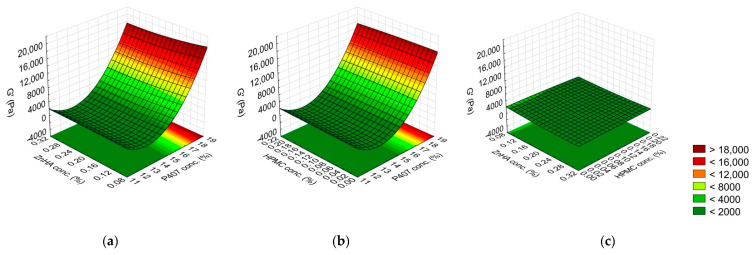
Effect of the composition on the G′ modulus: (**a**) P407 and ZnHA concentrations, (**b**) P407 and HPMC concentrations, (**c**) ZnHA and HPMC; (the 3rd factor is at 0 level in each case).

The G′ value was strongly determined by the P407 concentration (L, Q); however, the ZnHA concentration (L) and its interactions with other polymers (P407 (L) with ZnHA (L) and ZnHA (L) with HPMC (Q)) significantly decreases the gel strength (Figure 3, Table 4).

### 3.4. Mucoadhesion

Mucoadhesion is critical in terms of the residence time of eye drops on the ocular surface as it might reduce precorneal elimination. P407 has poor mucoadhesive properties [36], therefore its combination with good mucoadhesive polymers might be advantageous. Enhancement of mucoadhesion is expected from adding ZnHA and HPMC to the poloxamer-based formulations as these polymers are known as good bioadhesive excipients in ocular formulations [37,38]. The possible additive effect of the polymer combination was investigated by means of mucoadhesive force and work. Equations (7) and (8) describe the effect of the concentration of the polymers on the mucoadhesive parameters, namely peak force (y_4_) and work of adhesion (y_5_):

(7) $$y_{4}=1388.86+315.55x_{1}-66.81x_{1}{}^{2}-50.24x_{2}+78.97x_{1}{}^{2}x_{2}+114.97x_{1}x_{3}-38.72x_{1}{}^{2}x_{3}-74.29x_{2}{}^{2}x_{3}+55.13x_{2}{}^{2}x_{3}{}^{2}$$

(8) $$y_{5}=74.91+25.50x_{1}-5.30x_{1}{}^{2}-1.61x_{2}+2.63x_{3}-1.16x_{3}{}^{2}+1.95x_{1}x_{2}+1.63x_{1}{}^{2}x_{2}{}^{2}+3.88x_{1}x_{3}+1.19x_{2}x_{3}-1.95x_{2}{}^{2}x_{3}$$

**Figure 4:**
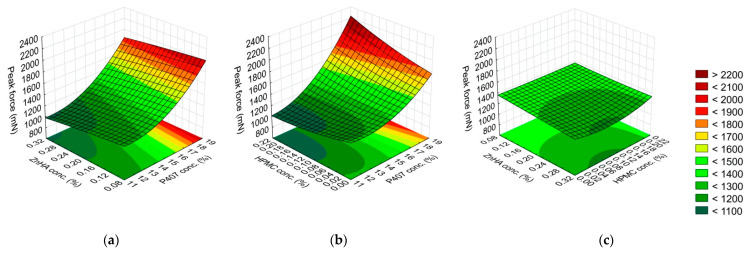
Effect of the composition on the adhesive force: (**a**) P407 and ZnHA concentrations, (**b**) P407 and HPMC concentrations, (**c**) ZnHA and HPMC; (the 3rd factor is at 0 level in each case).

In the case of the peak force values, the P407 concentration (L) was highly significant; furthermore, the interactions of P407 concentration with the mucoadhesive polymer concentration (P407 (Q) with ZnHA (L), and P407 (L) with HPMC (L)) were significant. All these factors were directly proportional to peak force. In contrast, the interaction of ZnHA concentration (Q) with HPMC concentration (L) significantly decreased the peak force values. Although the concentration of the mucoadhesive polymers (ZnHA and HPMC) alone was not significant, their interactions with P407 concentration were (Figure 4, Table 4). It suggests that both ZnHA and HPMC were important regarding the improvement of mucoadhesion.

**Figure 5:**
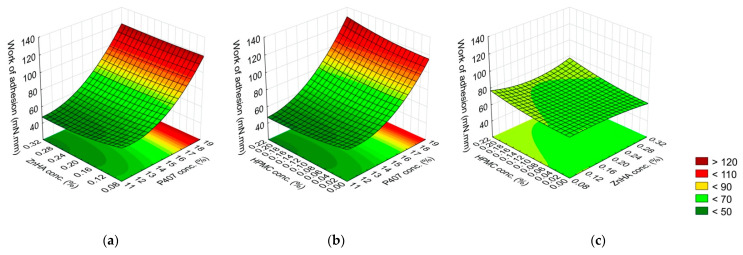
Effect of the composition on the adhesive work: (**a**) P407 and ZnHA concentrations, (**b**) P407 and HPMC concentrations, (**c**) ZnHA and HPMC; (the 3rd factor is at 0 level in each case).

The work of adhesion was determined by the P407 concentration (L, Q), HPMC concentration (L), and the interaction of P407 concentration (L) with HPMC concentration (L). The positive value of the coefficients of the linear factors shows that the higher concentration of P407 and HPMC was used in the formulations, the higher the work of adhesion value, thus the mucoadhesion was much stronger (Figure 5, Table 4).

### 3.5. In Vitro Drug Release and Corneal-PAMPA

Release efficiency ((Q)-6 h) shows the total percentage of DXM released from the gels and dissolved into the release medium in 6 h. The in vitro release rate (IVRR) provides information about the rate of drug release, i.e., the released amount of DXM per time unit (min) in the first 2 h of dissolution. It is desirable that the sample releases a relatively higher amount of API shortly after administration to reach the therapeutic concentration threshold, then maintains a continuous drug release for a long time. Furthermore, the released drug molecules should penetrate through the cornea to reach the site of action which can be considered a critical mechanism. In the literature, in vitro [24,39], ex vivo [39], and in vivo [40] tests are also presented in order to investigate the penetration. The corneal PAMPA method is used to mimic the upper layer of the lipophilic cornea, allowing a fast and reproducible permeability measurement of a large number of samples [24].

The effect of the P407, ZnHA, and HPMC concentration on the (Q)-6 h (y_6_), IVRR (y_7_), Pe (y_8_), and flux (y_9_) is described with the following equations (Equations (9)–(12)):

(9) $$y_{6}=67.40-1.89x_{1}+3.11x_{2}-3.48x_{2}{}^{2}-1.88x_{3}-2.40x_{3}{}^{2}+6.24x_{1}x_{2}+5.99x_{1}{}^{2}x_{2}-1.89x_{1}{}^{2}x_{2}{}^{2}-2.11x_{1}{}^{2}x_{3}+1.55x_{1}{}^{2}x_{3}{}^{2}+2.82x_{2}x_{3}+2.09x_{2}{}^{2}x_{3}{}^{2}$$

(10) $$y_{7}=1.47+0.12x_{1}{}^{2}+0.043x_{2}-0.096x_{3}{}^{2}+0.041x_{1}x_{2}-0.036x_{1}{}^{2}x_{2}{}^{2}-0.10x_{1}x_{3}-0.055x_{1}{}^{2}x_{3}+0.17x_{2}x_{3}+0.069x_{2}x_{3}{}^{2}$$

(11) $$y_{8}=78.13+23.15x_{1}-18.46x_{1}{}^{2}+8.45x_{3}+31.62x_{1}x_{2}-6.40x_{1}{}^{2}x_{2}+5.59x_{1}{}^{2}x_{2}{}^{2}-18.40x_{1}x_{3}{}^{2}-10.91x_{2}x_{3}-16.00x_{2}{}^{2}x_{3}+5.89x_{2}{}^{2}x_{3}{}^{2}$$

(12) $$y_{9}=2.46+0.66x_{1}-0.47x_{1}{}^{2}+0.21x_{3}+1.05x_{1}x_{2}-0.63x_{1}x_{3}{}^{2}-0.58x_{2}{}^{2}x_{3}$$

**Figure 6:**
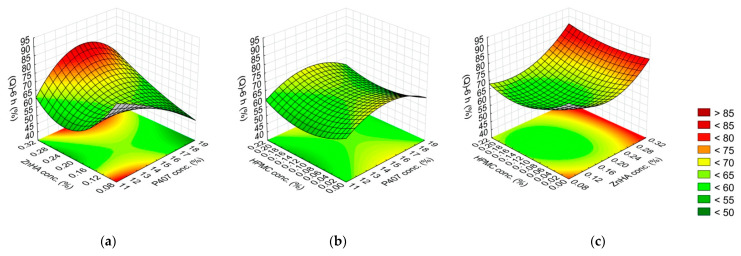
Effect of the composition on the released drug amount ((Q)-6 h): (**a**) P407 and ZnHA concentrations, (**b**) P407 and HPMC concentrations, (**c**) ZnHA and HPMC; (the 3rd factor is at 0 level in each case).

The released amount of DXM was determined by the ZnHA concentration (L, Q), the HPMC concentration (Q), and the interaction of HPMC (L) with the P407 concentration (L and Q). The linear factors and factor interactions were directly proportional to the released amount of DXM, whereas the increase in the concentration of the following factors, ZnHA (Q) and HPMC (Q), restrains the drug release. ZnHA highly significantly decreases the G′ value of the gels (Figure 6, Table 4). It might be associated with the higher drug release.

**Figure 7:**
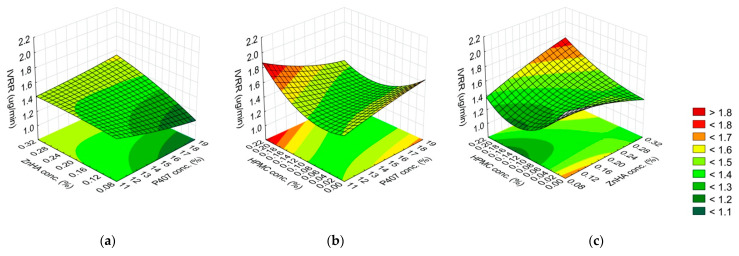
Effect of the composition on the IVRR: (**a**) P407 and ZnHA concentrations, (**b**) P407 and HPMC concentrations, (**c**) ZnHA and HPMC; (the 3rd factor is at 0 level in each case).

The IVRR of DXM from the samples was directly proportionally influenced by the concentration of P407 (Q) and the interaction of HPMC concentration (L) with the ZnHA concentration (L). In contrast, the HPMC concentration (Q) and its interaction with the P407 concentration (L) had the opposite effect (Figure 7, Table 4).

**Figure 8:**
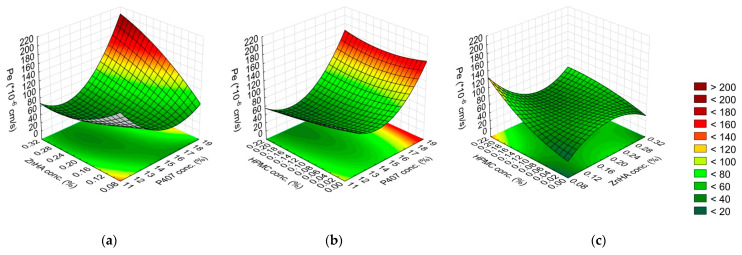
Effect of the composition on the permeability (Pe): (**a**) P407 and ZnHA concentrations, (**b**) P407 and HPMC concentrations, (**c**) ZnHA and HPMC; (the 3rd factor is at 0 level in each case).

**Figure 9:**
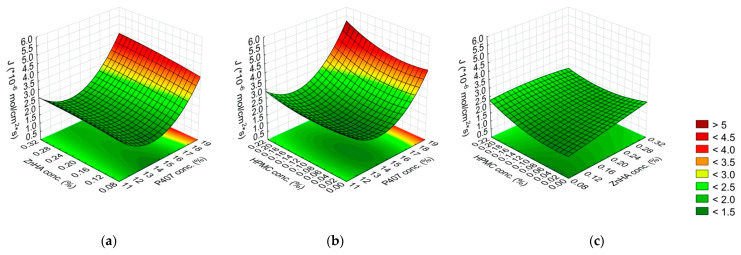
Effect of the composition on the flux (J): (**a**) P407 and ZnHA concentrations, (**b**) P407 and HPMC concentrations, (**c**) ZnHA and HPMC; (the 3rd factor is at 0 level in each case).

In the case of the permeability and flux of DXM in the corneal-PAMPA model, the same factors were significant. P407 (L) and its interaction with ZnHA (L) enhance the permeability and the rate of permeation as well. The other significant factors (P407 (Q), the interaction of P407 concentration (L) with HPMC concentration (Q), ZnHA concentration (Q) and HPMC concentration (L) interaction) produced the opposite effect. Increasing the P407 concentration increases the parameters of permeability; however, the negative coefficient of the non-linear factor suggests that the function has a minimum in the investigated concentration range (Figure 8 and Figure 9, Table 4).

**Figure 10:**
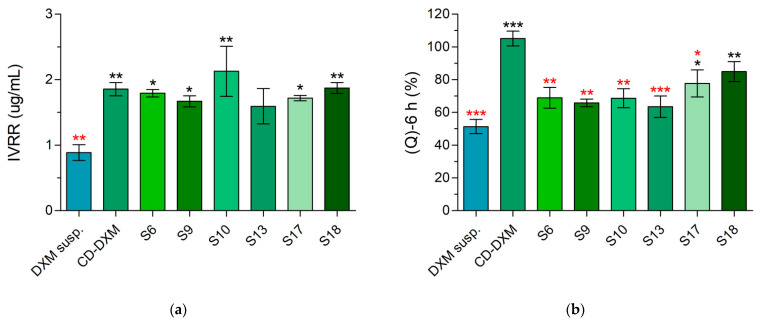
Comparison of in vitro drug release parameters of optimal formulations with DXM suspension. Statistical analysis: one-way ANOVA followed by Dunnett’s test. (* *p* ≤ 0.05 significant; ** *p* ≤ 0.01 very significant; *** *p* ≤ 0.001 highly significant difference. The difference from DXM suspension is indicated with black, the difference from CD-DXM solution is indicated with red). (**a**) Release efficiency of DXM suspension, CD-DXM solution and in situ gelling formulations. (**b**) In vitro release rate of DXM suspension, CD-DXM solution, and in situ gelling samples.

Based on the results of the experiments, some compositions (S6, S9, S10, S13, S17, S18) with desired rheological and mucoadhesive characteristics were chosen for further statistical analysis. In vitro drug release and corneal-PAMPA parameters of these samples were compared with DXM suspension (DXM susp.), and CD-DXM solution without polymers was also measured (Figure 10).

Out of the chosen formulations, S17 and S18 released a significantly higher amount of DXM than the suspension. These two samples had one thing in common: they both contained 15% P407, 0.3% ZnHA, and HPMC. Drug release from the in situ gelling samples was lower than the CD-DXM solution without any polymer except S18. In contrast, the IVRR was significantly higher, in the case of the eye drops, except for S13, whereas the IVRR value of the gels was similar to that of the CD-DXM solution. Although not all formulations showed an improvement in (Q)-6 h, the increase of IVRR indicates a change in the shape of the dissolution curve. The in situ gelling formulations did not release the total amount of DXM in 6 h; however, the dissolution profiles suggest that the drug release might have continued after 6 h (Figure S2). Changes in (Q)-6 h and IVRR indicate a better drug release profile of the gels than the suspension.

**Figure 11:**
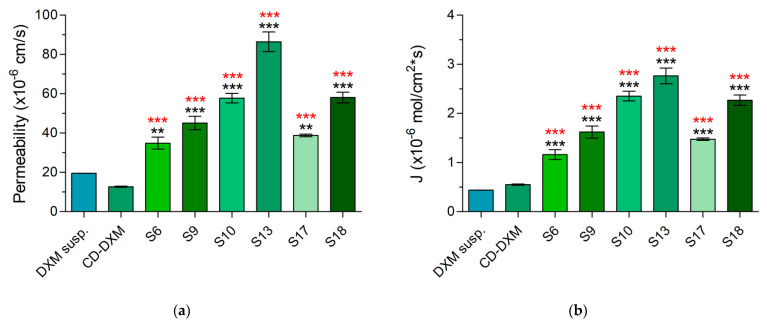
Comparison of corneal-PAMPA parameters of optimal formulations with DXM suspension. Statistical analysis: one-way ANOVA followed by Dunnett’s test. (** *p* ≤ 0.01 very significant; *** *p* ≤ 0.001 highly significant difference. The difference from DXM suspension is indicated with black, the difference from CD-DXM solution is indicated with red). (**a**) Permeability of DXM suspension, CD-DXM solution, and in situ gelling formulations. (**b**) Flux of DXM from DXM suspension, CD-DXM solution, and in situ gelling samples.

The permeability and the flux of the samples, without exception, were higher than those of the suspension (Figure 11). As these values were also significantly different from the CD-DXM solution, it is presumable that the increased permeability is due to the P407 content and not the inclusion complex.

## 4. Discussion

Although the bioavailability of topical preparations is low due to overflow, tear dilution, nasolacrimal drainage, or the API can be attached to the components of tears, it is more advantageous than those administered systemically because a lower dose is sufficient to achieve a therapeutic effect, so they cause little to no systemic effects, and the effect develops rapidly. To overcome these problems and improve bioavailability, reduction of elimination and increase of absorption are the main strategies.

The first aim of this work was to formulate in situ gelling eye drops that allow application as a liquid eye drop, then allow gelation to occur after distribution on the eye surface. The second aim of our work was to increase the mucoadhesion of the poloxamer containing eye drops and to increase the water solubility of the API.

CDs increase the water solubility and thereby enhance the bioavailability of poorly water-soluble APIs. Preformulation studies were performed to select the optimal CD derivate (HPBCD) and to determine the amount required to solubilize the therapeutic concentration of DXM (94.4 mM) based on the *K*_1:1_ value. Animal experiments and human studies [27,41,42] have shown that the aqueous solution of our selected HP*β*CD is well tolerated in ophthalmic preparations. Furthermore, CDs can reduce eye irritation by masking the irritant components or substituting certain irritant excipients in some formulations [14,15,43]. HPBCD is even used to solubilize indomethacin in a marketed eye drop (INDOCOLLYRE^®^). After the selection of the ideal CD, our aim was to produce a suitable in situ gelling matrix for this drug-CD inclusion complex.

Poloxamers are triblock copolymers consisting of hydrophilic and hydrophobic blocks that form micelles above the critical micelle concentration. Increasing the temperature lowers the critical micelle concentration. Upon further heating, the micelles aggregate forming a gel [3]. The advantages of using poloxamers in terms of low toxicity and good tolerability, but not outstanding in terms of mucoadhesion, are well known [3,36]; thus, our aim was to increase the mucoadhesion of eye drops in combination with other mucoadhesive polymers often used in ophthalmic formulations. When choosing the composition of the eye drops, safe ophthalmic application of each component was considered. Poloxamers have low toxicity; in a study, ocular liquid crystalline nanoparticles were evaluated in terms of cytotoxicity. The cubosomes were prepared with P407. Cell viability of rabbit corneal epithelial cells did not differ from the control, suggesting no toxicity [44]. Cellular studies of a composite system for ocular application (P407 21%, HPMC 1%) revealed a good cytocompatibility [45]. HPMC is often used in ophthalmic preparations, mainly as viscosity enhancer and gelling agent. According to the literature, ocular application of HPMC is safe [46]. According to a study, ocular films were prepared with 1.5% HPMC. It was proven that HPMC is biocompatible. MTT assay was performed on HeLa cells, and high cell viability was recorded even after 72 h [47]. Ophthalmic formulations, containing 5 mM HPBCD and 0.5% ZnHA, were tested on human corneal epithelial cells. After 30 min of treatment, cell viability was measured. The results showed no sign of cytotoxicity [48]. ZnHA (0.15%) is also used in a commercially available eye drop. Based on these results in the literature, the in situ gelling formulations of this study are expected to be non-cytotoxic.

The transmittance of the tested samples was not or only slightly reduced (>90%), and the refractive index values were within the physiological range (1.337–1.482 [32]), except for pure ZnHA and HPMC solutions. S27 was not transparent for harmful UV light (under 280 nm) but was for visible light that can be beneficial in ocular formulations. This phenomenon might be investigated more thoroughly in the future. Based on the results of the optical tests, the in situ gelling formulations presented in this study would probably not disturb the vision significantly.

The inverse proportionality between P407 concentration and gelling temperature is well-known in the literature [35]. However, the presence of other polymers can alter the gelation temperature and time determined by the P407 concentration, thereby affecting not only the gelation process but also the bioavailability of the eye drops. To explore this, the combined effect of the three polymers on gelation was investigated and the gel strength was characterized. In terms of gelling temperature and gelling time, 12–15% and 15–18% P407 was proved to be ideal, respectively. In a former study, it was shown that P407 was not able to form a gel in less than 17.5% concentration [49]. This seems to be contradictory to our findings; however, there is an explanation. There are multiple techniques of measuring the temperature of gelation. Two types of methods are commonly used by researchers: the tube inversion test and the one including a magnetic stirrer. The sample is gradually heated, and the gelation temperature is recorded when the preparation in the tube stops flowing when tilted, or when the magnetic stirrer stops moving. These visual methods are easy and fast, but the results are only approximate [50]. Rheological measurements are more accurate and sensitive to changes in the gel structure, and are also more suitable for detecting liquid-like gels than visual observation. Polymers can affect the orientation of the poloxamer chain during micelle formation and aggregation, thereby extending the gelling time. Polymers can also increase the viscosity, thus delaying gelation; however, they do not affect the gelation temperature as it is determined by only the concentration of P407. In contrast, it was previously observed that the increase of HPMC concentration (2–4%) reduced the gelling temperature of a P407-based in situ gel [51]. HPMC is known to form thermosensitive gels in the concentration range between 1–10% [52]. As our formulations contained a maximum of 0.2% HPMC, the insignificancy of it on the gelling temperature is understandable.

Although, gelling time is not as widely studied in the literature as gelling temperature, it is also important to consider optimizing in situ gelling formulations. On the one hand, when the gelation takes too long, the eye drops are less likely to resist the elimination mechanisms of the eye. On the other hand, immediate gelation hampers the distribution of the eye drop on the eye surface, thereby a discomfort in the eye is possible. All formulations containing 15% P407 and some of the 12% P407-containing ones had ideal gelling time (<5 min), while the 18% P407 resulted in such rapid gelation, that might prevent proper distribution on the eye surface. The gelling time of the formulations can be comparable with other studies [53,54].

The strength of the gel formed is clearly determined by P407, but it can also be seen that the gel strength decreased in combination with other polymers. Presumably, the polymers interfere with the micelle network, weakening the formation of secondary bonds. In this aspect, ZnHA weakened the gel strength the most.

Mucoadhesion is important in terms of bioavailability due to increased residence time. P407 is preferred for use in ophthalmic formulations; however, there are polymers with better mucoadhesive properties (e.g., hyaluronic acid [37], HPMC [55]), with which we combined poloxamer. In terms of peak force, the P407 appears in greater amounts at the interface where chemical bonds are formed between the polymer and the mucin. Since peak force depends on the formation of chemical bonds between the polymer and the mucin [56], P407 concentration being the most important factor is not surprising here. Other than chemical bonds, physical entanglement and interpenetration of the polymeric chains and mucin determines the work of adhesion value [56]. The results of this study show that not just P407, but other polymers, especially HPMC, play a significant role in the interpenetration of polymer chains, thus determining mucoadhesion. In a previous study, the statistical analysis of in situ gels, containing two types of poloxamers and HPMC (0.5–1.5%), showed that HPMC was significant in terms of mucoadhesion [38]. The combination of P407 and hyaluronic acid was also proved to be beneficial in ocular mucoadhesive drug delivery systems [57].

Prolonged drug release was expected from the gel structure, so drug release was experimentally investigated. IVRR shows the drug release rate of the first 2 h of dissolution. This parameter is important as a prompt effect is expected from these eye drops. The cumulative drug release at 6 h provides information on the release over longer time. Regarding IVRR, the interaction of P407 and ZnHA is the most significant, and (Q)-6 h is mainly influenced by ZnHA. This can be explained by the gel strength lowering effect of ZnHA. Wei et al. [58] reported that combining poloxamers with high molecular weight sodium hyaluronate also weakened the gel strength, and consequently increased the drug release. In contrast, Mayol et al. [57] recorded the opposite using low molecular weight hyaluronic acid and explained this phenomenon with the difference in the molecular weight. Many researchers reported sustained drug release from thermosensitive in situ gels [35,59,60]. The in vitro dissolution study of an in situ gel, containing methazolamide, showed similar results to ours, as 44.9% of the API was released from the gel in the first hour, then the release became prolonged after 1 h [35].

The drug release properties were also compared with conventional suspension and CD-DXM solution. For the gels, the IVRR was not different from the CD-DXM solution, so a similar prompt effect can be expected. In contrast, the 6-h values suggest that the drug release becomes prolonged in the second stage of dissolution. In view of these results and the increased mucoadhesion, sustained drug release can be predisposed. Significantly higher IVRR values compared with the suspension show more favorable release through CD complexation. For (Q)-6 h, certain in situ gelling compositions (S17, S18) showed much more favorable values compared with both the suspension and the CD-DXM solution.

Topically applied medications should reach the site of action by transcorneal penetration into the interior segment of the eye. The formulation and the composition can influence the penetration as well. CD-DXM complexes diffuse from the matrix to the interface, where the complex dissociates and only the free drug can penetrate through the cornea. For this reason, the stability constant of the complex may be critical although this value may be affected by the polymers. P407 might increase corneal permeation by further increasing the water-solubility of DXM. Additionally, it can remove the phospholipids from the epithelial cell membrane by complexation but does not damage the membrane; furthermore, P407 can relax the cell junctions of the epithelium resulting in the increased influx of DXM through the cornea [61]. In our work, P407 enhanced drug penetration. The more P407 in the eye drop, the more viscous it is, which may slow down the release of the drug, so there will be less free drug that can penetrate, but the permeation enhancing effect of P407 is advantageous. ZnHA also increases the permeability due to increased drug release from the gel matrix. In PAMPA assays, the concentration of P407 in the layer around the membrane may be higher, which may increase penetration. The experimental results support that poloxamer enhances the penetration, as higher permeability values were obtained for the gels compared with not only the DXM suspension but also the CD-DXM solution.

## 5. Conclusions

Our goal was to produce an ideally gelling mucoadhesive matrix for the CD-DXM complex, which was achieved in addition to solubility. We also successfully increased the mucoadhesion with the polymers (ZnHA and HPMC) and achieved much better drug release and penetration properties with the polymer combination compared with the suspension and with the CD-DXM solution.

## Figures and Tables

**Table 1:** Values and levels of the independent variables.

Parameter	Code	−1	0	+1
P407 (%)	x_1_	12	15	18
ZnHA (%)	x_2_	0.1	0.2	0.3
HPMC (%)	x_3_	0	0.1	0.2

**Table 2:** *K*_1:1_ and CE values of the DXM-CD derivate inclusion complexes and the slope of the linear function.

CD Derivate	Slope	*K*_1:1_ (M^−1^)	CE
HPBCD	0.2910	1006.7	0.4104
HPGCD	0.5500	2997.8	1.2222
DIMEB	0.5491	2987.1	1.2178
SBEBCD	0.2360	757.9	0.3090

**Table 3:** Refractive index values of the compositions.

Composition	Refractive Index
P407 (18%)	1.3569
ZnHA (0.3%)	1.3332
HPMC (0.2%)	1.3331
P407 (18%) + ZnHA (0.3%)	1.3576
P407 (18%) + HPMC (0.2%)	1.3573
P407 (18%) + ZnHA (0.3%) + HPMC (0.2%)	1.3578
S27	1.3611

**Table 4:** Results of the experiments (mean values) of the samples for the factorial design.

Sample	P407 (%)	ZnHA (%)	HPMC (%)	Gelling Temp. (°C)	Gelling Time (min)	G′ (Pa)	Peak Force (mN)	Work of Adhesion (mN·mm)	(Q)-6 h (%)	IVRR (μg/min)	Pe (×10^−6^ cm/s)	J (×10^−6^ mol/cm^2^·s)
S1	12	0.1	0	35.6	5.4	285.2	1136.5	58.8	87.0	1.40	42.8	1.60
S2	12	0.1	0.1	35.7	5.9	171.3	1261.2	52.7	69.5	1.40	142.6	4.91
S3	12	0.1	0.2	34.6	5.4	137.8	1379.2	61.9	65.9	1.40	108.7	3.18
S4	12	0.2	0	36.6	5.5	168.7	1238.0	53.5	62.4	1.35	33.3	0.93
S5	12	0.2	0.1	31.8	4.4	168.2	1061.8	50.3	60.9	1.09	103.4	2.90
S6	12	0.2	0.2	33.8	4.0	227.7	904.4	50.8	69.0	1.79	34.9	1.16
S7	12	0.3	0	35.4	5.4	217.5	1253.7	52.0	65.3	1.27	60.1	1.62
S8	12	0.3	0.1	33.5	6.4	187.7	867.5	49.2	60.0	1.32	34.7	1.06
S9	12	0.3	0.2	30.5	4.7	321.0	958.2	47.4	65.9	1.67	45.1	1.62
S10	15	0.1	0	28.4	0.9	1718.2	1282.3	64.9	68.7	2.13	57.8	2.35
S11	15	0.1	0.1	27.5	1.0	1746.4	1175.8	66.9	64.9	1.39	31.1	0.91
S12	15	0.1	0.2	27.0	0.8	2042.7	1240.7	69.9	51.6	1.41	78.9	2.45
S13	15	0.2	0	28.7	1.1	1334.0	1313.5	68.7	63.5	1.59	86.5	2.76
S14	15	0.2	0.1	27.9	1.0	1553.7	1422.8	68.5	62.5	1.46	57.1	1.76
S15	15	0.2	0.2	27.6	2.4	1455.2	1233.9	72.6	56.5	1.51	43.9	1.37
S16	15	0.3	0	23.5	1.1	1045.4	1424.0	67.0	89.0	1.51	29.6	1.18
S17	15	0.3	0.1	28.5	1.0	1133.4	1221.4	64.8	77.7	1.72	38.8	1.48
S18	15	0.3	0.2	28.4	1.2	1425.9	1383.7	67.3	85.0	1.87	58.1	2.27
S19	18	0.1	0	23.9	0.4	1282.3	1789.3	103.0	69.4	1.59	59.7	1.92
S20	18	0.1	0.1	23.6	0.4	13,765.7	1666.2	102.9	45.3	0.94	18.1	0.44
S21	18	0.1	0.2	23.6	0.4	12,147.3	2072.6	111.3	65.9	1.31	152.9	4.49
S22	18	0.2	0	23.8	0.4	12,172.7	1706.4	100.9	65.1	1.62	138.6	4.83
S23	18	0.2	0.1	23.5	0.4	11,996.7	1841.6	102.1	66.5	1.23	94.8	2.56
S24	18	0.2	0.2	23.6	0.4	11,804.0	1673.5	99.8	58.6	1.24	102.2	2.61
S25	18	0.3	0	23.9	0.4	12,106.3	1283.4	88.9	67.6	1.32	130.4	3.75
S26	18	0.3	0.1	23.7	0.4	11,462.3	1682.0	102.9	70.5	1.51	159.6	4.82
S27	18	0.3	0.2	23.2	0.4	11,970.3	2025.6	123.9	74.6	1.56	166.1	5.50

## Data Availability

Data are contained within the article or Supplementary Materials.

## Supplementary Materials

The following supporting information can be downloaded at: https://www.mdpi.com/article/10.3390/gels8090561/s1, Figure S1: Phase solubility curves of the different CD-DXM complexes.; Figure S2: In vitro DXM release from suspension, CD solution, and complex polymer composition.; Figure S3: Transmittance of the compositions; Table S1: Results of the experimental and the adjustments.
