# Supplementary material for: Design and Optimization of In Situ Gelling Mucoadhesive Eye Drops Containing Dexamethasone
Source: Gels. 2022 Sep 2;8(9):561. doi: 10.3390/gels8090561 (PMC9498616; doi:10.3390/gels8090561)
Supplement: Supplementary file 1 [file gels-08-00561-s001.zip › gels-1893488-SI.pdf]

gels

Supplementary materials

Design and Optimization of In Situ Gelling Mucoadhesive Eye Drops Containing Dexamethasone

Boglárka Szalai [1], Orsolya Jójárt-Laczkovich [1], Anita Kovács [1], Szilvia Berkó [1], György Tibor Balogh [2,3], Gábor Katona [1] and Mária Budai-Szűcs [1,*]

[1] Institute of Pharmaceutical Technology and Regulatory Affairs, Faculty of Pharmacy, University of Szeged, Eötvös Str. 6, H-6720 Szeged, Hungary
[2] Department of Pharmacodynamics and Biopharmacy, Faculty of Pharmacy, University of Szeged, Eötvös Str. 6, H-6720 Szeged, Hungary
[3] Department of Chemical and Environmental Process Engineering, Budapest University of Technology and Economics, Műegyetem Quay 3, H-1111 Budapest, Hungary
* Correspondence: budai-szucs.maria@szte.hu; Tel.: +36-6254-5573

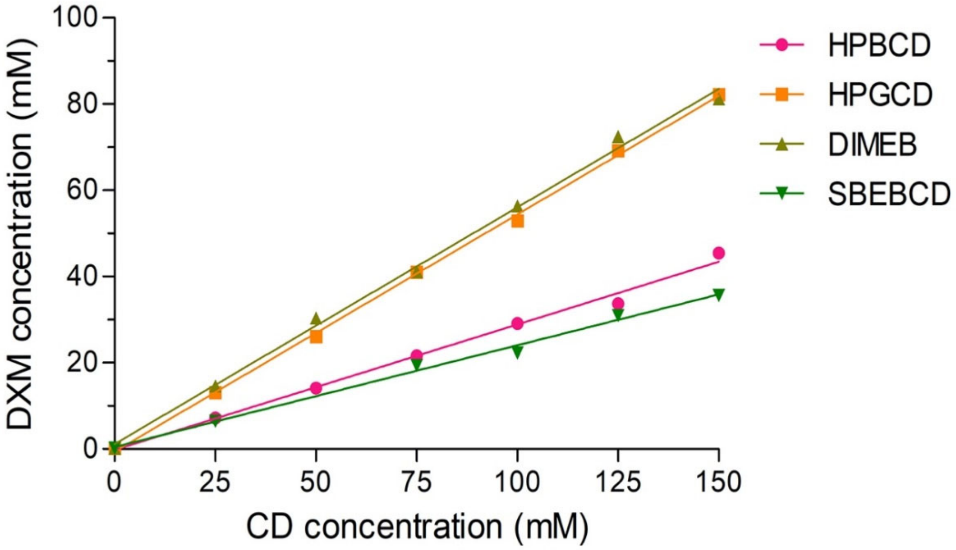

Figure S1. Phase solubility curves of the different CD-DXM complexes.

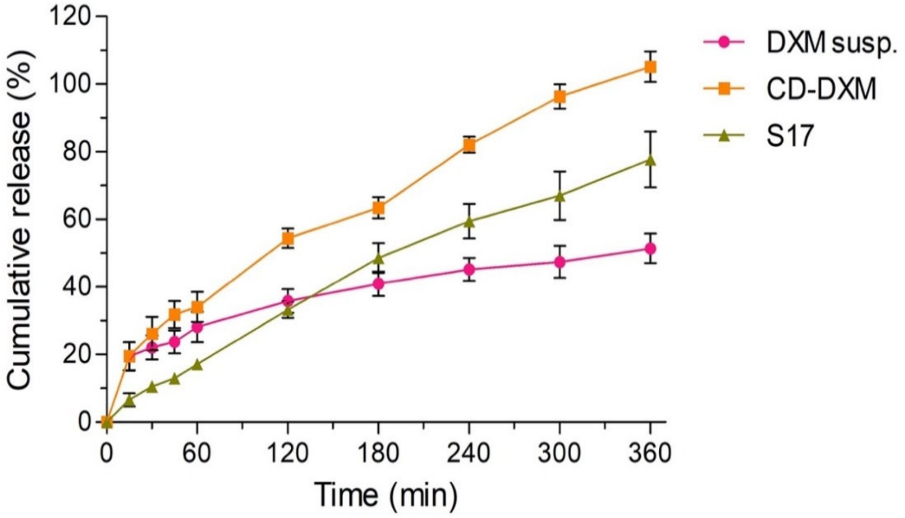

Figure S2. *In vitro* DXM release from suspension, CD solution and complex polymer composition.

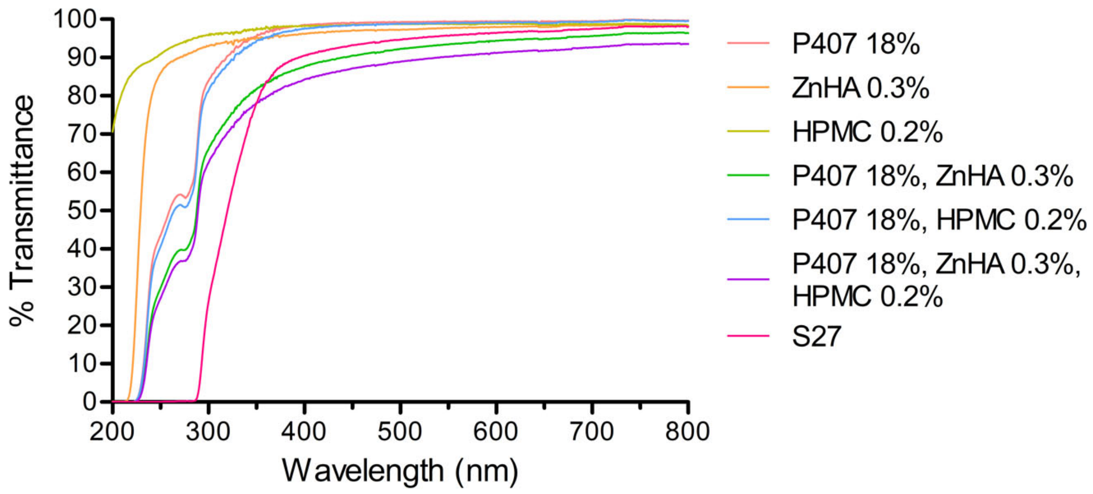

Figure S3. Transmittance of the compositions.
